# Components of the item selection algorithm in computerized adaptive testing

**DOI:** 10.3352/jeehp.2018.15.7

**Published:** 2018-03-24

**Authors:** Kyung (Chris) Tyek Han

**Affiliations:** Graduate Management Admission Council, Reston, VA, USA; Hallym University, Korea

**Keywords:** Algorithms, Computers, Computerized adaptive testing, Probability, Test taking skills

## Abstract

Computerized adaptive testing (CAT) greatly improves measurement efficiency in high-stakes testing operations through the selection and administration of test items with the difficulty level that is most relevant to each individual test taker. This paper explains the 3 components of a conventional CAT item selection algorithm: test content balancing, the item selection criterion, and item exposure control. Several noteworthy methodologies underlie each component. The test script method and constrained CAT method are used for test content balancing. Item selection criteria include the maximized Fisher information criterion, the *b*-matching method, the *a*-stratification method, the weighted likelihood information criterion, the efficiency balanced information criterion, and the Kullback-Leibler information criterion. The randomesque method, the Sympson-Hetter method, the unconditional and conditional multinomial methods, and the fade-away method are used for item exposure control. Several holistic approaches to CAT use automated test assembly methods, such as the shadow test approach and the weighted deviation model. Item usage and exposure count vary depending on the item selection criterion and exposure control method. Finally, other important factors to consider when determining an appropriate CAT design are the computer resources requirement, the size of item pools, and the test length. The logic of CAT is now being adopted in the field of adaptive learning, which integrates the learning aspect and the (formative) assessment aspect of education into a continuous, individualized learning experience. Therefore, the algorithms and technologies described in this review may be able to help medical health educators and high-stakes test developers to adopt CAT more actively and efficiently.

## Introduction

The emergence and advancement of modern test theory (e.g., item response theory [IRT]) and the rapid deployment of new computing technologies have completely changed how educational tests are designed, delivered, and administered. One of the most important examples is computerized adaptive testing (CAT). As its name implies, the CAT test form is adaptively assembled on the fly as the test taker answers each test item. Because CAT selects and administers items at the difficulty level that is most relevant to each test taker’s ability level, the test length is usually much shorter than a typical linear version of a test. At the same time, CAT administration retains (or even improves) the precision of test score estimation—in other words, CAT greatly improves measurement efficiency.

CAT has been introduced to a variety of high-stakes testing operations, including medical health licensing examinations in the United States such as the American Society for Clinical Pathology Board of Certification Examinations, the National Council Licensure Examinations (NCLEX-RN exam), National Registry of Emergency Medical Technicians, and the North American Pharmacist Licensure Examination. To adopt CAT for use in other licensing examinations not only in the United States, but in other countries around the world, it is essential to understand the basic processes of CAT administration, of which the item selection algorithm is the most critical component. The purpose of this review is to present knowledge and techniques regarding the 3 components of the conventional CAT item selection algorithm: test content balancing, the item selection criterion, and item exposure control. It is hoped that this review will help opinion leaders of test-providing institutions to understand CAT implementation and administration.

## Three (iterative) processes of CAT administration

**Fig. 1.**
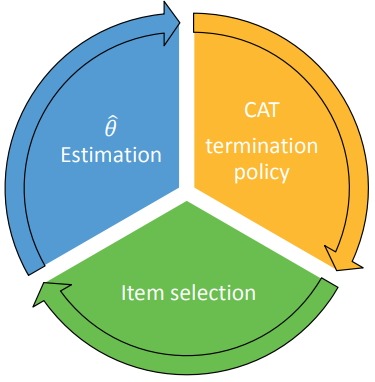
Three (iterative) processes of computerized adaptive testing (CAT).

CAT administration involves 3 key processes that iterate for each test item administration, as shown in the illustration in Fig. 1.

The first process determines the latest $\hat{\theta }$ (i.e., the ability estimate). At the beginning of CAT, there are no observed response data from which $\hat{\theta }$ is estimated, so the $\hat{\theta }$ value is initialized at the expected value, which is often the average score. After the response data are collected for one or more items, $\hat{\theta }$ is computed and updated using, for example, the maximum likelihood estimation (MLE) or Bayesianbased methods [1] such as the modal a posteriori [2] or expected a posteriori (EAP) [3]; methods. Test developers often prefer the MLE method because it does not have the estimation biases that Bayesianbased methods usually exhibit due to their use of an informative prior. The MLE method, however, is often unusable in an early-stage CAT administration due to its inability to handle special response patterns such as all correct or all incorrect responses. To overcome this issue, it is not unusual for several different $\hat{\theta }$ estimation methods to be used during CAT in operational settings. For example, in the early stage of CAT administration, when the number of item responses is small and the chance of having all correct or all incorrect responses is high, the EAP method is used. As the CAT progresses toward or reaches the end of a test, the final $\hat{\theta }$ is estimated using the MLE method. A modification of MLE, called MLE with fences (MLEF), was recently proposed by Han [4]. The literature suggests that MLEF results in unbiased estimation just like MLE, but is also capable of handling special response patterns that a typical MLE cannot.

The second CAT process involves the evaluation of test progress against the CAT termination rules that are defined as part of the test specification. In many CAT programs, the test length is fixed for all test takers, and the CAT administration is terminated once the number of items reaches the fixed test length. This is preferable if the goal is to make the test-taking experience, especially test time and workload, equivalent across test takers. In CAT, however, the length of a test and its termination can be adaptive, too, based on other termination rules and policies. For example, some CAT test programs aim to ensure that the precision of the score estimation is as parallel as possible across test takers while shortening the test length as much as possible. In such an example, the CAT termination rule can be defined such that the test administration finishes once the standard error of estimation (SEE) for a test score reaches a target. If the progress and status of the CAT do not meet the termination criteria, then the CAT is set to move on to the third process.

**Fig. 2.**
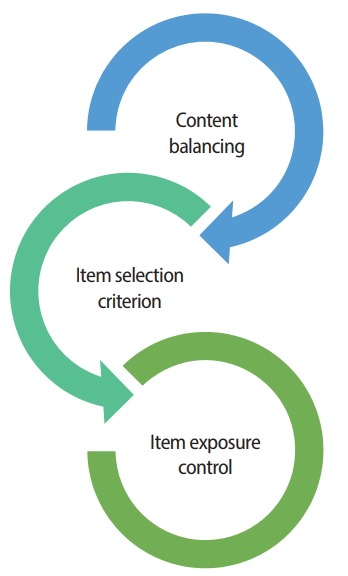
Three components of the computerized adaptive testing item selection algorithm.

The third process involves the selection and administration of a new item given the latest interim $\hat{\theta }$ from the first process. The item selection process is the core function of CAT, and is where test construction and delivery actually happen. The item selection process involves 3 key components: (1) content balancing, (2) the item selection criterion, and (3) item exposure control, as illustrated in Fig. 2. Many CAT algorithms handle these 3 components separately for ease of implementation, but some CAT approaches combine or handle some or all of the 3 item selection components together at the same time. This paper introduces several of the most widely used or noteworthy methodologies for each of the CAT item selection components, followed by a discussion of other approaches.

## Content balancing

In most test form constructions, it is common for a test form to have more than 1 content area from which items are selected, and it is important for CAT to ensure that the composition of test content is parallel across all test takers, even while it adaptively alters the test difficulty for each individual.

One of the most widely used approaches for balancing test content involves the use of a test script. A test script specifies which items are eligible for item selection based on content area given the item administration sequence. For example, if we were to administer 5 test items from 3 different content areas— say, 1 item from content area 1, 2 items from content area 2, and 2 items from content area 3—the test script would look like the one shown in Table 1. If, for test security reasons, it is preferable to differentiate the sequence of test content across test takers, one could employ multiple test scripts and randomly select one of them for use when CAT testing starts. The test script method is easy to understand, straightforward to implement, and greatly simplifies the item pool assembly process since each item belongs to a single content category and content categories are mutually exclusive (i.e., no overlap of content areas is allowed). As a result, the test script method is widely used in various CAT applications. However, 2 critical shortcomings are associated with the test script method. First, because the test script specifies a content area for each sequence of item administration, each test script is specific to the test length. If, for example, a CAT is of a variable length instead of a fixed length, the test script method may not achieve a completely consistent content balance across test takers. Second, because the test script method requires that all items belong to a single content category, the method may not be directly applicable if there are multiple layers in the content structure. For example, if there are 2 layers of content categories (content area and type) as shown in Table 2, one would need to reclassify all items into 1 of the 6 possible content combinations (3 content areas by 2 content types). The test script method often is still practical for content balancing if there are 2 or 3 content layers after reclassifying items. If there are 4 or more layers, however, the number of available items in each mutually exclusive category across all layers can become very small, making the test script method impractical.

Another popular method for content balancing in CAT is the constrained CAT (CCAT) method proposed by Kingsbury and Zara [5]. In CCAT, the percentage of items administered thus far from each content area is first evaluated against the content specification/ target. After that, the items from a content area that show the greatest proportional difference compared with the content specification/ target are identified as eligible for item selection for the next CAT administration. Because CCAT evaluates the test content composition by percentage instead of the actual count of items, it can be used in both fixed-length CAT and flexible-length CAT. In addition, multiple layers of content balancing can be easily handled by implementing CCAT as many times as the number of content layers. One undesirable property of CCAT, however, is that the sequence of test content areas during CAT administration is always the same across all test takers.

To address this downside of the CCAT method, Leung et al. [6] proposed a modified CCAT (MCCAT) method. Unlike CCAT, MCCAT considers all items eligible for item selection except those from content areas where the maximum number of items has already been reached. With the MCCAT approach, the sequencing of content areas during CAT is much less predictable. One could also expect this approach to improve the adaptiveness of CAT, since MCCAT tends to have a greater number of eligible items to select from in comparison with CCAT.

When test content is specified by a range of numbers or percentages instead of exact numbers or percentages, the penalty function approach proposed by Segall and Davey [7] or its variations such as the weighted penalty model [8] can be considered as alternative options for content balancing.

Other approaches exist that perform both content balancing and item selection simultaneously instead of handling them separately. These approaches will be introduced later in this paper.

## Item selection criteria

Once test items are determined to be eligible based on the content specification in the content balancing component of the CAT algo-rithm, these eligible items are then evaluated for selection as the next item to be administered. An extensive array of item selection criteria has been developed in the test measurement field, but only a very few are used in actual CAT implementation. An introduction to a few noteworthy item selection criteria is presented below.

### Maximized Fisher information

One of the most well-known item selection criteria—and probably the oldest criterion—involves selecting an item with the maximized Fisher information (MFI) at a given $\hat{\theta }$ (i.e., the more recent interim score estimate) based on test items previously administered to a test taker [9]. The Fisher information (a.k.a., item information function [IIF]) for item *i* can be computed as

(1) $$I_{i}\left(\theta \right)=\frac{\left[P_{i}^{'}\left(\theta \right)\right]}{P_{i}\left(\theta \right)Q_{i}\left(\theta \right)'}$$

where *P_i_* (θ) is the probability of a correct response from a person at a given θ, *Q_i_* (θ)= 1-*P_i_* (θ), and *P´_i_* (θ) is the first derivative of *P_i_* (θ). If items are calibrated with a 2-parameter logistic (2PL) IRT model, *P_i_* (θ) can be computed as

(2) $$P_{i}\left(\theta \right)=\frac{1}{1+e^{-Dai\left(\theta -b_{i}\right)}}$$

and its Fisher information for item *i* (Equation 1), therefore, reduces to

(3) $$I_{i}\left(\theta \right)=D^{2}a^{2}P_{i}\left(\theta \right)Q_{i}\left(\theta \right)$$

where *D* is the scaling constant of 1.702.

For example, if there are 4 eligible items to select from Table 3 and

**Fig. 3.**
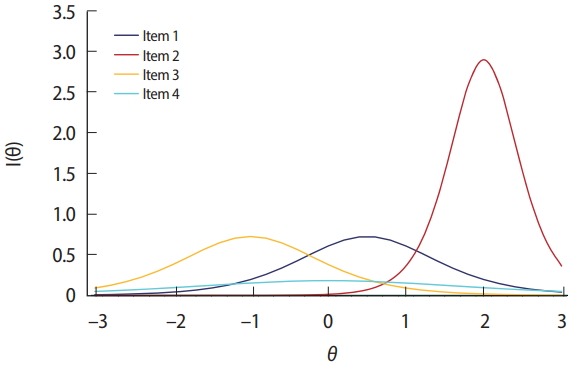
Example of computerized adaptive testing item information functions.

**Fig. 4.**
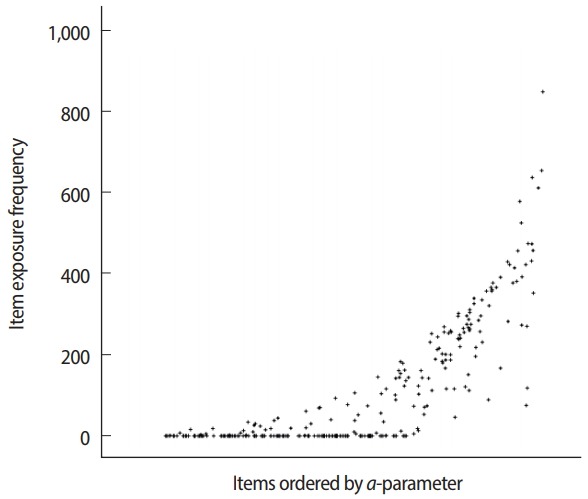
Example of typical item usage/exposure patterns with the maximized Fisher information criterion.

$\hat{\theta }$ is 0.5, item 1 would be the best choice according to the MFI criterion because item 1 exhibits the largest IIF value (Fig. 3). If $\hat{\theta }$ equals 1.5, item 2 would be the best item according to the MFI criterion, so item 2 would be selected and administered. The MFI criterion for CAT item selection is easy to understand and implement, and is also known to result in the maximum test information function for each CAT session because it always selects the item expected to show the largest Fisher information value. The MFI criterion is rarely used in actual operational CAT applications, however, because of its ‘greedy’ tendency in item selection. For example, as shown in Fig. 3, item 4 does not show a high IIF peak due to its low *a*-parameter value (= 0.5). Additionally, there are other items that exhibit a higher IIF at any given θ between −3 and 3. Therefore, there is no chance that item 4 would be selected and used under the MFI criterion in this example. Fig. 4 displays an example of a typical item usage and exposure pattern with the MFI criterion. In this example, CAT administers 30 out of 300 items in the pool based solely on the MFI criterion. The figure clearly shows a pattern of excessive use of items with higher aparameter values, as well as a pattern of infrequent use of items with lower *a*-parameter values. The ‘greedy’ nature of MFI item selection imposes serious threats to test security and creates issues with item pool utilization, and thus has led to the development of other item selection criteria and item exposure methods.

### Difficulty matching criterion

The item selection approach that uses the item difficulty matching (a.k.a., *b*-matching) criterion evaluates the distance between the interim $\hat{\theta }$ and the *b*-parameters of all eligible items and selects the item with minimal distance. This approach is commonly used when test items are calibrated with a 1-parameter logistic (1PL) model or Rasch model since items exhibit the most information when their difficulty is closest to the θ value. In fact, the ‘*b*-matching approach’ essentially results in the same item selection pattern as the MFI ap-proach when a 1PL or Rasch model is used. The *b*-matching criterion is often used with items calibrated with 2PL or 3PL models as well, since, unlike the MFI criterion, it does not demonstrate the ‘greedy’ item-selection pattern that selects only higher *a*-parameter values.

### Interval information criterion

In the MFI criterion, a test item’s information is computed at a point of interim $\hat{\theta }$ . In the early stages of CAT, for example, after only 2 or 3 items are administered, one can expect the estimation error associated with $\hat{\theta }$ to be quite large. Therefore, selecting an item based on the inaccurate point estimate of θ in early stages of CAT can result in less than optimal item selection. To address this issue, Veerkamp and Berger [10] proposed the interval information criterion, whereby each eligible item is evaluated by the IIF averaged across the confidence interval of an interim $\hat{\theta }$. The mathematical expression of the interval information criterion for item *i* is

(4) $$\int_{\theta ={\hat{\theta }}_{L}}^{{\hat{\theta }}_{R}}I_{i}\left[\theta \right]d\theta$$

where $\hat{\theta }$L and $\hat{\theta }$R indicate the confidence interval of *θ*.

### Weighted likelihood information criterion

In addition to the interval information criterion, Veerkamp and Berger [10] also proposed the weighted likelihood information (WLI) criterion for item selection. In the WLI criterion, the information function is summed throughout the *θ* scale, weighted by the likelihood function after the items administered thus far. With the WLI criterion, the item to be selected is item i, which results in the maximized value of

(5) $$\int_{\theta =-\infty }^{\infty }L\left(\theta ;\chi_{m-1}\right)I_{i}\left[\theta \right]d\theta$$

where L(*θ; x_m-1_*) is the likelihood function of the response vector *x_m-1_* after the (*m*-1)th item administration.

Based on their simulation studies, Veerkamp and Berger [10] reported that the item selection results using the interval information criterion and the WLI criterion showed some improvement in CAT performance over the MFI criterion for extreme *θ* values.

### a-Stratifi ation method

**Fig. 5.**
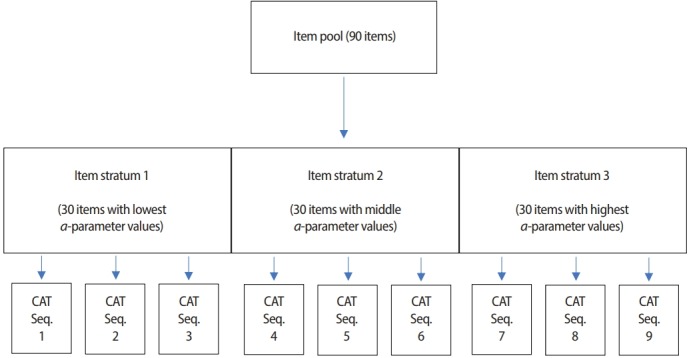
Example of the a-stratifi tion method. CAT, computerized adaptive testing; Seq, sequence.

As mentioned earlier, when the MFI criterion is used for item selection in the early stage of CAT, it often selects items that are far from optimal due to the inaccurate $\hat{\theta }$. Chang and Ying [11] proposed a different approach, whereby items with higher *a*-parameter values are reserved for use in the later stage of CAT by stratifying all items in the item pool by *a*-parameter values. For example, if an item pool has 90 eligible items and a total of 9 items need to be selected and administered, as shown in Fig. 5, the items can be grouped into 3 item strata by their *a*-parameter values (30 items in each item stra-tum). At the beginning of CAT (the first 3 item administrations, for example), CAT selects and uses an item with a difficulty level that is closest to $\hat{\theta }$ among the items in item stratum 1. As CAT progresses into its later stages, items are selected from an item stratum with higher *a*-parameter values. The overall performance of the *a*-stratification method has been proven to be solid as long as the item pool is optimally designed—meaning that it does not show the ‘greedy’ item selection pattern seen in the MFI criterion—while minimizing its tradeoff in measurement efficiency.

A potential issue with the *a*-stratification method is that in realworld applications, it is common to observe a moderate positive correlation between *a*- and *b*-parameters. In other words, items with higher *a*-parameter values tend also to have higher *b*-parameter values. Because of that, stratifying an item pool by an item’s *a*-parameter value could unintentionally result in items being stratified by their *b*-parameter value as well. For example, the item stratum with the highest *a*-parameter values is likely to end up with items whose *b*-parameter values are also much higher than other item strata with lower *a*-parameter values. This could lead to a serious shortage of items with specific difficulty levels within each item stratum. To address this issue, Chang et al. [12] proposed a modification called astratification with *b*-blocking. In the modified version, items are first stratified by their *b*-parameter values, and then the items from each *b*-parameter stratum are grouped by their *a*-parameter values to construct item strata that are based on *a*-parameters while being balanced in the *b*-parameter.

The *a*-stratification method (and its modification) generally yields stable performance, striking a balance between CAT measurement efficiency and overall item pool utilization, as long as the item pool is large and optimally designed. If the item pool is small, however, or if there are many content categories and test constraints, the actual number of eligible items within each item stratum could be extremely small. Under such circumstances, the CAT’s level of adaptability with this item selection method could suffer a serious downturn. Additionally, because the *a*-stratification method determines which item stratum to select an item from according to the CAT process, it is not usable when the test length is not fixed.

### Efficiency balanced information criterion

The efficiency balanced information (EBI) criterion was developed by Han [13] to better utilize items with lower *a*-parameter values, as in the *a*-stratification method, but with no need to stratify the item pool. One component of the EBI criterion involves evaluating the expected item efficiency (EIE), which is defined as the level of real-ization of an item’s potential information at an interim $\hat{\theta }$. If item ishows maximum potential information at $\theta_{i}^{*}$, the EIE at interim $\hat{\theta }$ after the *j*-th item administration is computed as

(6) $$EIE=\frac{I_{i}\left[{\hat{\theta }}_{j}\right]}{I_{i}\left[\theta_{i}^{*}\right]}$$

where $\theta_{i}^{*}$ is equal to *b_i_* when using either a 1PL or 2PL model. In the EBI criterion, the EIE (Equation 6) is assessed across a *θ* interval. The width of the *θ* interval for the item efficiency (IE) evaluation is determined by the SEE (ε) and set to 2 SEEs from $\hat{\theta }$ after the *j*-th item administration $\left({\hat{\theta }}_{j}+2\varepsilon_{j}\right)$. Therefore, the IE value for item *i* is computed as

(7) $$IE_{i}\left[{\hat{\theta }}_{j}\right]=\int_{{\hat{\theta }}_{j}-2\varepsilon_{j}}^{{\hat{\theta }}_{j}+2\varepsilon_{j}}\frac{I_{i}\left[\theta \right]}{I_{i}\left[\theta_{i}^{*}\right]}d\theta .$$

It should be noted that when εj is large (e.g., during the early stage of CAT administration), an item with a lower *a*-parameter will result in a larger IE value if all other conditions are the same among items. Items with a lower *a*-parameter tend to show greater efficiency at a wider range of *θ*.

The EBI criterion evaluates not only the IE but also the item information expected at $\hat{\theta }$ during the item selection procedure. Like IE, the item information for each item is also evaluated within $\left({\hat{\theta }}_{j}+2\varepsilon_{j}\right)$. This is nearly identical to the interval information criterion that Veerkamp and Berger [10] proposed. By combining the IE component (Equation 7) and the information component, the EBI criterion eventually looks for an item resulting in the maximized EBI, which is calculated as:

(8) $$EBI_{i}\left[{\hat{\theta }}_{j}\right]=\int_{{\hat{\theta }}_{j}-2\varepsilon_{j}}^{\hat{\theta }j+2\varepsilon_{j}}\frac{I_{i}\left[\theta \right]}{I_{i}\left[\theta_{i}^{*}\right]}d\theta +\int_{{\hat{\theta }}_{j}-2\varepsilon_{j}}^{\hat{\theta }j+2\varepsilon_{j}}I_{i}\left[\theta \right]d\theta =\left(1+\frac{1}{I_{i}\left[\theta_{i}^{*}\right]}\right)\int_{{\hat{\theta }}_{j}-2\varepsilon_{j}}^{{\hat{\theta }}_{j}+2\varepsilon_{j}}I_{i}\left[\theta \right]d\theta$$

With this criterion, items with lower *a*-values tend to have a better chance of being selected at the beginning of CAT, whereas items with higher *a*-values occur more frequently in the later stages.

### Kullback-Leibler information criterion

Chang and Ying [14] developed the global information approach, which uses the moving average of Kullback-Leibler information (KLI) to select items [15,16]. The KLI for any *θ* for the *i*-th item with response *X_i_* is defined as

(9) $$K_{i}\left(\theta ∥\theta_{0}\right)=P_{i}\left(\theta_{0}\right)\log\left[\frac{P_{i}\left(\theta_{0}\right)}{P_{i}\left(\theta \right)}\right]+\left[1-P_{i}\left(\theta_{0}\right)\right]\log\left[\frac{1-P_{i}\left(\theta_{0}\right)}{1-P_{i}\left(\theta \right)}\right]$$

where *P_i_(*θ**_0_) is the probability that a random test taker at proficiency level *θ*_0_ answers the item correctly. The moving average of KLI is then calculated and used as the item selection criterion, as follows,

(10) $$K_{i}\left(\theta_{0}\right)=\int_{\theta_{0}-\xi }^{\theta_{0}+\xi }K_{i}\left(\theta ∥\theta_{0}\right)d\theta$$

where ξ specifies the range of the moving average. The resulting determination of ξ could be ambiguous, so Chang and Ying [14] proposed $c\;/\;\sqrt{m}$ as a reasonable choice for ξ, with constant *c* selected ac-cording to a specified coverage probability and with *m* being thenumber of items administered thus far. Chang and Ying [14](1996) found that replacing the MFI criterion with the KLI criterion often reduced the biases and mean-squared errors of proficiency estimation when the test length was short (*m* < 30) or when the CAT administration was in its early stage, where the $\hat{\theta }$ often contains a large estimation error.

## Item exposure control

Unlike linear tests whose test items are designed for a single use during 1 testing event, CAT reuses all items in the item bank/pool over time. However, some items may be selected and used too frequently. Such excessive exposure of items to a test population couldchange a person’s test-taking behavior regarding those compromised items. For example, if a test taker obtained prior knowledge of the compromised test items and their correct answers, he or she could likely respond based on memorization of the items rather than on his or her true problem-solving skills. Unintended changes in testtaking behavior due to excessive item exposure could seriously threaten the test’s fairness and validity. In many high-stakes exams, maintaining proper item exposure is one of most important considerations for ensuring test security. It should be noted that for some CAT applications, such as medical diagnostic questionnaires, personality measures, and adaptive learning tools, controlling item exposure is not necessary.

Item exposure control is the last of the 3 components of the CAT item selection algorithm (Fig. 2). Once eligible items are identified in the first component (content balancing), and an optimal item is selected in the second component (item selection), then, in the third component (exposure control), some random factors are introduced to the item selection process to prevent excessive item use. The following section introduces a few widely used exposure control methods.

### Randomesque

Kingsbury and Zara [5] proposed employing the randomesque method to keep the best item from being solely (or excessively) used in CAT administration. Instead of selecting a single best item, this method is designed to select multiple best items based on the item selection criterion. After that, one of the best items is randomly administered. The randomesque method may not be highly effective in limiting the maximum item exposure rate to a target rate, but it can prevent the same item from being used repeatedly for test takers with similar proficiency levels. This method and its variations are widely used in CAT practice as a sole method (or as an additional means) for controlling item exposure as well as for enhancing overall item pool utilization.

### Sympson-Hetter method

In the probabilistic approach developed by Sympson and Hetter [17], the probability P(A) that an item will be administered is differentiated from the probability P(S) that the item will be selected based on the item selection criterion. In other words, the Sympson-Hetter (SH) method introduces the conditional probability P(A|S) that the selected item will actually be administered. In order to keep the P(A) at a desirable target level, the P(A|S) that results in the target P(A) is derived from iterative simulations. Once the P(A|S) is computed for each item in the item pool, it is treated as the exposure parameter in the actual item-selection process. During CAT administration, all eligible items are ordered by the choice of item selection criterion. Start-ing from the best item, the item exposure parameter is compared against a randomly generated value between 0 and 1 (following a uniform distribution). If the random value is smaller than the expo-sure parameter, the item is administered; otherwise, the process proceeds to the next best item. This process is repeated until an item is finally administered. It is important to note that the computed exposure parameters are pool-specific; in other words, the exposure parameters should be recomputed whenever there is a change in the item pool, even in a single item.

### Unconditional multinomial method

The unconditional multinomial (UM) method is similar to the SH method in that it computes exposure parameters using iterative simulations [18]. What differentiates the UM method from the SH method is that the UM method first forms a multinomial distribution from each item’s P(A|S) and then compares the distribution to a random value to determine which item to actually administer.

### Conditional multinomial method

The SH and UM methods are useful for controlling the overall exposure for each item, but they do not guarantee the desired exposure rate within each group of test takers of similar proficiency. In the conditional multinomial (CM) method [19,20], each item has multiple exposure parameters that correspond to each proficiency group. The exposure parameters are computed separately for each proficiency group during the simulations. Once the exposure parameters are computed, the exposure parameter for the corresponding proficiency group based on the (interim) $\hat{\theta }$ estimate is used to form a multinomial distribution. The rest of the procedure is the same as the UM method.

### Fade-away method

Today’s computer networking technology makes it possible for main computer servers and client computers (i.e., test terminals) in test centers to communicate before, during, and/or after CAT administration to reconfigure a variety of test information, including item usage. Complete item usage information maintained in the main server can be updated regularly by the client computers during or after each CAT administration via the online network. In addition, each client computer can access updated item usage information from the server just before the start of the next test administration. Such network technology enables the CAT system to use near real-time item exposure information for exposure control, precluding the need to predict item exposure by other means, such as using the SH method [17], which involves iterative simulations.

In the fade-away (FA) method [21], the item selection criterion value for each eligible item in the pool is inversely weighted by the ratio between the updated actual exposure rate and the target exposure rate. For example, with the MFI criterion displayed in Equation 1, the CAT system looks for an item that maximizes

(11) $$I_{i}\left[{\hat{\theta }}_{m-1}\right]\frac{U_{i}}{C}$$

where *C* is the absolute item usage limit (of the first exposure con-trol component) and *U_i_* is the item usage for the life of item *i*. With this new method, rarely used items are expected to be promoted more frequently, and excessively used items are likely to “fade away” from the item selection. This method can be especially useful and effective in CAT with cloud-based systems.

## CAT using automated test assembly approaches

Earlier sections of this report explained each of the 3 separate components of CAT item selection algorithms (Fig. 2). As noted, a majority of operational CAT programs currently implement and run those 3 item selection components separately. There are other CAT approaches, however, that construct adaptive test forms and handle the content balancing and item selection components simultaneously. These CAT approaches view and formulate the content balancing component and item selection component as the constraints and objective, respectively, of a mathematical programming model such as mixed integer programming (MIP), an optimization method often used for automated test assembly (ATA) practices [22-26].

The shadow-test approach (STA) offers a framework for iterative ATA performed on the fly given the latest interim $\hat{\theta }$ for CAT [27,28]. In this framework, either minimizing the deviation of the test information function (TIF) from the TIF target or maximizing the TIF itself at a single or multiple evaluation points on the *θ* scale is set to be an objective for the MIP, and the test content balancing and other test specifications are formulated as constraints. For example, if the goal is to implement an STA that is equivalent to a 10-item-long CAT using the MFI item selection criterion (see Equation 1) with the content balancing scenario shown in Table 2, then the MIP model can be expressed as:

(12) $$\mathrm{maximize}\;\sum_{i=1}^{I}I_{i}\left({\hat{\theta }}^{\left(g-1\right)}\right)\;(\mathrm{objective})$$

(13) $$\mathrm{subject}\;\mathrm{to}\sum_{i=1}^{I}\chi_{i}=n=10,\;(\mathrm{test}\;\mathrm{length})\sum_{i=1}^{I}\chi_{i}C_{1i}=10\times 0.4,\;(\mathrm{content}\;\mathrm{type}\;1,\;\mathrm{pure})\sum_{i=1}^{I}\chi_{i}C_{2i}=10\times 0.6,\;(\mathrm{content}\;\mathrm{type}\;2,\;\mathrm{real})\sum_{i=1}^{I}\chi_{i}C_{3i}=10\times 0.3,\;(\mathrm{content}\;\mathrm{area}\;1,\;\mathrm{arithmetic})\sum_{i=1}^{I}\chi_{i}C_{4i}=10\times 0.4,\;(\mathrm{content}\;\mathrm{area}\;2,\;\mathrm{algebra})\sum_{i=1}^{I}\chi_{i}C_{5i}=10\times 0.3,\;(\mathrm{content}\;\mathrm{area}\;3,\;\mathrm{geometry})$$

where *i* = {1, 2, 3, …, *I*}, *I* is the number of items in the item pool, *n* is the test length, *x_i_* is a binary variable indicating whether item *i* is included in the constructed test form (1 if included and 0 if not included), *g* is the number of items administered so far, and *C_1i_*, *C_2i_*, *C_3i_*, *C_4i_*, and C5i are binary (0 or 1) identifiers for item i for each content type/area (for example, if *C_1i_* is 1, the item *i* content type is “pure”).

Once the MIP model is set, the MIP solver does most (if not all) of the heavy lifting of test form construction for STA-CAT. Unlike all previously mentioned CAT approaches, where a single item is eventually selected and administered during each cycle of the CAT process (Fig. 1), in CAT with STA, a complete test form given $\hat{\theta }$ is constructed each time. The complete test form always satisfies all content and other constraints, which is its huge advantage over other approaches. In STA-CAT, each item can have multiple content categories and/or multiple layers of content categories. The MIP solver is not restricted to selecting 1 item at a time, which could degrade the level of CAT adaptiveness. Because the MIP solver is required to compute a completely new full test form in real time whenever $\hat{\theta }$ is updated, however, the computational resources requirement for STA-CAT can be extremely high. Thus, it is often necessary to tune the solver and constraints to make STA-CAT operationally feasible.

Stocking and Swanson [29] also proposed another approach to CAT within a linear-programming framework. They focused on developing an MIP model that, unlike STA-CAT, could tolerate violations of content constraints to enable the CAT system to be more robust against possible item selection failures even when, in the view of STA-CAT, there would be no feasible solution for meeting all content constraints. In their method, called the weighted deviation model (WDM), Stocking and Swanson [29] treated the content constraints as part of the object function, where the violations of the content constraints are to be minimized as the object. The IIF is also considered in the WDM-CAT as one of the weighted deviations from the target. Even though the WDM-CAT can be implemented using an MIP solver to find the optimal solution, as with STA-CAT, Stocking and Swanson instead proposed the use of a heuristic algorithm [30], in which items are evaluated and selected one at a time based on the object function of WDM as opposed to items for a whole test form. Using the WDM with the heuristic algorithm, the computational load for CAT administration is much lower than STA-CAT, but un-like STA-CAT, each test form based on WMD is not guaranteed to meet all content constraints.

## Choosing an optimal CAT approach

When it comes time to implement a test program, it is common for some less experienced practitioners to view this as a contest of selecting among different CAT methods to find the ‘best’ method before even asking themselves whether CAT is the right solution. As with any other test construction task, the development of a CAT-based test requires the consideration of many important factors, including the purpose of the test, skills to be measured, nature of the test population, time limit, test time window, test-taking experience, test security, development time and cost, test volume, and communication with test users and stakeholders.

A key question to ask before delving into the details of CAT design decisions is whether CAT is the right design for the test program. Although CAT has joined the mainstream of test industry methods and is used in many psychological and educational applications, it is not necessarily the silver bullet for all testing applications. For exam-ple, if the goal of a test is to render a pass/fail decision for test takers, then using a fixed test form or linear-on-the-fly test (LOFT) developed to maximize the TIF at the cut score (of pass/fail decision) can be more efficient than CAT. If the desired test outcome is simply to classify test takers into one of multiple categories, then a multistage testing (MST) design may be a reasonable alternative to CAT. If maximizing score reliability across a wide-ranging score scale is the goal, however, then CAT is usually the best option compared with less adaptive options such as MST or LOFT.

CAT is usually best suited for large-scale assessments with huge test volumes and continuous or multiple test windows. If the test volume is very small, however (e.g., fewer than 100 test takers per year), it will take a long time or likely be impossible to build up a usable item bank. Even with a large test volume and response data to calibrate items, CAT requires significant lead time for the item bank to grow large enough to address test security concerns through item exposure control. Therefore, it is not unusual for a test program to start initially with a non-CAT fixed test form and later transition to CAT-based test administration once the item bank is well established.

### Interactions between item selection criteria and exposure control

Selecting the right method for each of the 3 components of the item selection process—content balancing, the item selection criterion, and item exposure control—is not straightforward and cannot and should not be considered separately for each of these 3 components because of the unique interactions among them.

**Fig. 6.**
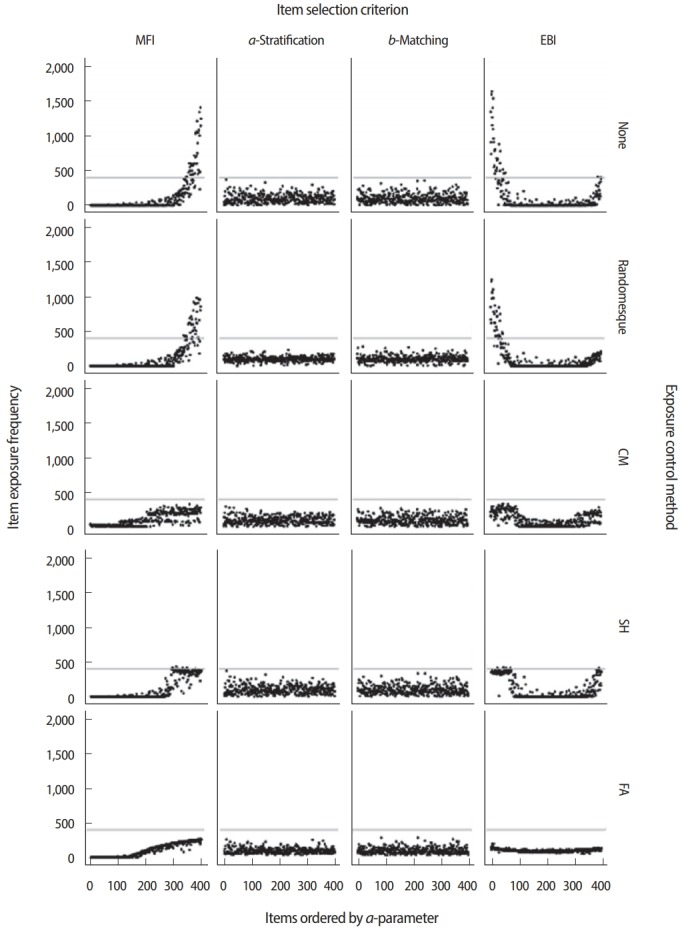
Item usage and exposure count with different item selection criteria and exposure control methods. MFI, maximized Fisher information; EBI, efficiency balanced information; CM, conditional multinomial; SH, Sympson-Hetter; FA, fade-away.

**Fig. 7.**
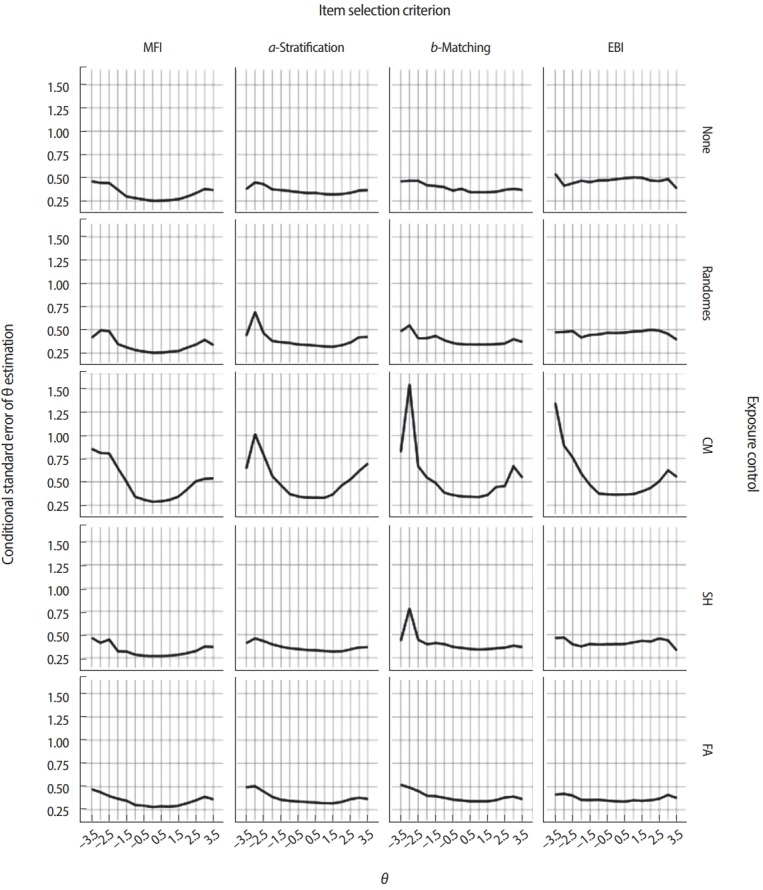
Conditional standard error of estimation across θ values. MFI, maximized Fisher information; EBI, efficiency balanced information; CM, conditional multinomial; SH, Sympson-Hetter; FA, fade-away.

Figs. 6 and 7 demonstrate how different combinations of item selection criteria and item exposure control methods could result in sharply different CAT performance and item usage patterns. The illustrations demonstrate 20 possible combinations using 4 different item selection criteria (MFI, *a*-stratification, *b*-matching, and EBI) paired with 5 different exposure control methods (none, randomesque, SH, CM, and FA). Fig. 6 displays the item usage/exposure patterns by an item’s *a*-parameter values under each condition, and Fig. 7 shows the conditional standard error of *θ* estimation (CSEE). Except for the item selection criterion and item exposure control method, all other test conditions were identical: *θ* values were generated for 2,000 simulees following a standard normal distribution, each simulee was administered 20 items, and each item pool contained 400 items. As shown in Fig. 6, when the MFI criterion was used with no item exposure control, more than half of items in the pool were not used at all, while items with higher *a*-parameter values were used excessively. Because the MFI criterion always selects items that will maximize the information function, the CSEE was the smallest with the MFI criterion compared with other criteria in the absence of any exposure control (Fig. 7). The *a*-stratification and *b*-matching criteria showed even item usage regardless of an item’s *a*-parameter value, even without any additional exposure control. When the EBI criterion was used with no item exposure control, it tended to excessively select items with lower *a*-parameter values. As a result, the CSEE was noticeably larger than the cases using other item selection criteria.

When the studied item selection criteria were paired with the randomesque method for exposure control, the maximum item exposure was slightly reduced across all item selection criteria (Fig. 6) without any severe impact on CSEE (Fig. 7), although the maximum item exposure was still concerningly large for the MFI and EBI cases. When the SH item exposure control method was used, it was strictly mandated that items be used no more than 20% of the time (500 out of 2,000 simulees) with the MFI and EBI criteria. The SH method showed no meaningful change with the *a*-stratification and bmatching criteria because those criteria never showed a maximum exposure rate larger than 0.2 in the first place. When the FA method was used to control item exposure, the EBI criterion showed the most even item usage pattern among all 4 criteria, although its CSEE was consistently low throughout the *θ* intervals. The MFI criterion with the FA item exposure method also showed a significantly lower maximum exposure rate, without necessarily leading to the promotion of underused items in item selection. When the CM method was used to control item exposure for each of 6 different *θ* groups at the rate of 0.2, the item usage pattern was similar to those seen with the SH method (Fig. 6), but there were serious surges of CSEE at *θ* < −1.5 and *θ* > 1.5 (Fig. 7), regardless of the item selection criterion chosen. The increased CSEEs at extreme *θ* values were the result of the CM method’s tight control of the item exposure rate, which limited the number of items with either very low or very high b-parameter values in the pool. When the CM method limited the maximum usage of items with very low or very high b-parameter values, items lacking the optimal difficulty level were forced into use as an alternative, eventually leading to a dramatic increase in CSEE.

On the whole, the examples in Figs. 6 and 7 illustrate how interactions not only between the item selection criterion and item exposure control method, but also with the item pool, can create different outcomes in CAT performance and behavior. If the item pool is deep, with an extremely large number of items across all content categories and difficulty levels, such interactions tend to have a minimal impact on the CSEE and item exposure rate. Most real-life test situations, however, operate with a limited number of items in an item pool along with several content and other constraints, which usually result in some level of tradeoff between item selection optimality and exposure control level. In general, the measurement efficiency of CAT tends to decrease as item exposure control becomes stricter, with the exception of the EBI criterion used with the FA exposure control method (Fig. 7). The tradeoff between measurement efficiency and item exposure control is almost impossible to estimate accurately by analytical means, which is why conducting simulation studies to evaluate the tradeoff and other CAT performance metrics is critically important in deciding upon a CAT design.

### Conventional 3-component approach versus ATA-based approaches

When comparing the CAT approaches that employ separate procedures for each of the 3 item selection components (Fig. 2) with the more holistic, ATA-based approaches such as the STA and WDM,there is no clear winner—each has its own advantages and disadvantages. The STA often can yield a more optimally selected item set than conventional item selection processes where items are selected one at a time and only from the eligible items for a particular sequence (due to the content balancing component). Thus, in situa-tions involving multiple constraints, a short test, and a limited item pool, the ATA-based approaches can be more effective. However, conventional CAT methods are often more cost-effective to implement (e.g., they do not require an MIP solver) and, more important, are straightforward in terms of identifying and troubleshooting issues regarding each of the item selection components. In the examples shown in Figs. 6 and 7, it becomes obvious that the CSEEs can surge at some *θ* ranges when the MFI method is used with the CM item exposure control given that particular item pool. Based on the identified issue, one can arrive at possible solutions to address it by tackling the potential causes from each item selection component. For example, relaxing some item content balancing parameters mightpromote the inclusion of more optimal items given the item selection criterion. Alternately, adjusting the CM item exposure control setting to have a slightly higher exposure rate target or to have fewer *θ* groups for conditional control may help reduce the CSEE. It is also possible that increasing the size of the item pool by adding more items with extreme *b*-parameter values could ultimately resolve the issue. Under the conventional CAT algorithm with 3 item-selection components, different approaches to address the issue can be easily tried separately at each component level. In contrast, when the ATAbased approaches such as the STA fail to create a test form, it is often difficult to understand what exactly is causing the failure of test assembly.

The computing resources requirement is also an important factor to consider when deciding between the STA and conventional CAT. The STA usually requires a high level of computing power with significant memory or a powerful cloud system with a stable internet connection. In contrast, for most conventional CAT methods, modern personal computers or communication devices such as smartphones and tablet PCs can handle CAT delivery without a constant need for an internet connection. The size of the item pool and the test length also should be taken into consideration. With the STA, if the item pool is large (for example, 2,000 to 5,000 items) and/or the test length is extremely long (for example, 100 to 500 items), the solver could fail to find an optimal test form within a reasonable time (e.g., less than 1 second). For most conventional CAT methods, the effect of pool size and test length on processing time is minimal in practice.

## Conclusion

The fundamental logic behind CAT (especially under the IRT framework) was developed more than 50 years ago, but CAT has only gained wide acceptance in the educational and psychological measurement field and in various applications across large-scale test programs in the last 20 years. Extensive research in CAT is actively underway. Topics currently under investigation include new item selection methods, new ATA methods, new CAT designs that improve the test-taking experiences (e.g., allowing response review and change), live item calibration during CAT, new multidimensional CAT methods, new diagnostic CAT methods, and test security measures specialized for CAT administration. In addition, the logic of CAT is now being adopted in the field of adaptive learning, which integrates the learning aspect and the (formative) assessment aspect of education into a continuous, individualized learning experience, and is gaining traction throughout the K-12 educational system. It is a truly exciting time to be involved in the CAT field. Understanding the key elements of CAT reviewed and discussed in this paper is critically important, and, at the same time, efforts to stay current with the new and upcoming CAT research are also very important for measurement experts.

## Figures and Tables

**Table 1.** Example of a test script for content balancing

Item administration sequence	Content area
1	2
2	2
3	3
4	3
5	1

**Table 2.** Example of 2 layers of content for a mathematics exam

Content type (layer 2)	Content area (layer 1)			
	Arithmetic	Algebra	Geometry	Total
Type 1 (pure mathematical expressions)	Content category	Content category	Content category	40
	1 (12)	2 (16)	3 (12)	
Type 2 (real-world context)	Content category	Content category	Content category	60
	4 (18)	5 (24)	6 (18)	
Total	30	40	30	100

Values are presented as number (%) or %.

**Table 3.** Example of 4 eligible items in a 2-parameter logistic model

Item ID	*a*-parameter	*b*-parameter
1	1.0	0.5
2	2.0	2.0
3	1.0	–1.0
4	0.5	0.0

## Supplementary materials

Supplement 1. Audio recording of the abstract.
